# Evaluation of Periorbital Tissues in Obstructive Sleep Apnea Syndrome

**DOI:** 10.4274/tjo.galenos.2020.35033

**Published:** 2020-12-29

**Authors:** Irmak Karaca, Ayşe Yağcı, Melis Palamar, Mehmet Sezai Taşbakan, Özen K. Başoğlu

**Affiliations:** 1 Ege University Faculty of Medicine, Department of Ophthalmology, İzmir, Turkey; 2 Ege University Faculty of Medicine, Department of Chest Diseases, İzmir, Turkey

**Keywords:** Obstructive sleep apnea syndrome, periorbital tissue, floppy eyelid, eyelid laxity, eyelash ptosis

## Abstract

**Objectives:**

To evaluate periorbital tissue alterations including eyelid laxity and eyelash ptosis in patients with obstructive sleep apnea syndrome (OSAS).

**Materials and Methods:**

Based on polysomnography, 96 eyes of 48 patients with moderate/severe OSAS (Group 1) and 44 eyes of 22 patients with simple snoring (Group 2) were enrolled. Comprehensive eye examination along with eyelid laxity measurements including vertical and anterior distraction, presence of dermatochalasis, interpalpebral distance, and levator function were assessed. The presence and severity of eyelash ptosis were also noted.

**Results:**

The mean ages of Group 1 and Group 2 were 49.9±11.4 (range: 26-67) and 50.6±8.9 (range: 27-69) years, respectively (p=0.557). The mean vertical and anterior distraction distances in Group 1 (13.3±4.1 [range, 6-27] mm and 7.4±2.1 [range, 3-13.5] mm, respectively) were significantly higher than in Group 2 (p<0.05). Dermatochalasis and eyelash ptosis were found to be significantly more frequent in Group 1 (52.1% and 81.3%, respectively). The severity of eyelash ptosis was also higher in OSAS (p<0.05). No significant difference in interpalpebral distance or levator muscle function was detected.

**Conclusion:**

In patients with severe OSAS, eyelid laxity was more prominent and eyelash ptosis was more frequent and severe.

## Introduction

Obstructive sleep apnea syndrome (OSAS) is a sleep disorder characterized by recurring episodes of apnea-hypopnea (AH) lasting 10 seconds or longer and a decrease in oxygen saturation.^1^ The prevalence of OSAS is 2-4% for symptomatic cases, although it has been estimated that a large proportion of the population may have undiagnosed OSAS.^2,3^ The prevalence is up to 21-90% among patients referred to sleep outpatient clinics.^4^ With the increase in obesity in recent years, the prevalence of OSAS is rising rapidly.^5^ As awareness increases among society and healthcare workers, more people are presenting to sleep clinics with typical symptoms such as snoring, witnessed apnea, and excessive daytime sleepiness. However, the disease can still be easily overlooked.^6^

In terms of pathophysiological changes in OSAS, intermittent hypoxia, cyclic desaturations, and elevated catecholamine levels affect the sleep-wake cycle.^7^ Resultant changes such as systemic hypertension, atherosclerosis, endothelial dysfunction, insulin resistance, and autonomic dysfunction may result in comorbidities such as coronary heart disease, stroke, congestive heart failure, and even death.^8,9^ The possibility that OSAS may impact ocular vascular health via these mechanisms and cause or exacerbate ocular problems remains a concern and the subject of ongoing research.^10^ In the literature, OSAS has been associated with floppy eyelid syndrome (FES)^11,12^, glaucoma^13^, ischemic optic neuropathy^14^, papilledema^15^, nocturnal lagophthalmus^16^, central serous chorioretinopathy^17^, and retinal vein occlusion.^18^

This study aimed to evaluate periorbital tissue alterations such as eyelid laxity and eyelash ptosis in patients with OSAS.

## Materials and Methods

This single-center, prospective, cross-sectional study was performed between March 2016 and May 2017 in the ophthalmology department of Ege University Faculty of Medicine (EUFM). The study included a total of 70 patients with no previous OSAS diagnosis, among whom 48 and 22 patients were diagnosed as having OSAS and simple snoring, respectively, according to their AH index (AHI) score in polysomnography (PSG) evaluation in the sleep laboratory of the EUFM chest diseases department. Patients with AHI ≥15 were classified as moderate or severe OSAS (Group 1; 39 men and 9 women) and patients with AHI <5 were classified as simple snoring (Group 2; 12 men and 10 women).

Exclusion criteria were: 1) previous or current nasal continuous positive airway pressure therapy; 2) history of intraocular/extraocular surgery, ocular trauma or chemical injury; 3) presence or treatment history for any eye disease other than refractive error such as glaucoma, dry eye syndrome, thyroid ophthalmopathy; 4) history of contact lens use; 5) history of chronic steroid use; 6) smoking; and 7) diabetes mellitus or thyroid dysfunction detected in systemic evaluation.

Ethics approval was obtained from the Institutional Review Board of EUFM (16.02.2016, no: 16-1/6). The study was conducted in accordance with the principles of the Declaration of Helsinki and the patients were informed about the study scope and the evaluations involved.

Following PSG evaluation, all patients were referred for detailed ophthalmological examination including best corrected visual acuity, intraocular pressure measurement, anterior segment examination, funduscopy, and evaluation of cup/disc ratio, as well as evaluations of interpalpebral fissure height and levator function, eyelid laxity assessment with upper lid vertical distraction distance^19^ and upper lid anterior distraction distance^20^, presence and degree of eyelash ptosis^21^, loss of eyelash alignment, presence of FES^11,12^, and presence of dermatochalasis. The researcher who performed the ophthalmological examinations (I.K.) was blind to the patients’ PSG results.

Interpalpebral distance was defined as the maximum distance between upper and lower lids with the eyes in primary gaze. Levator function was measured as the distance traveled by the edge of the upper lid between downward and upward gaze while applying pressure to the brow to block frontalis muscle action. Moreover, during ophthalmological examination, the upper eyelid was grasped from the pretarsal skin and pulled vertically by manual traction to evaluate whether the eyelid folded easily and upper lid vertical distraction distance was recorded for both eyes as the distance between the palpebral rim of the upper lid and the pupil center after applying manual vertical traction on the upper eyelid.^19^ In both eyes, upper eyelid anterior distraction was determined, with the palpebral rim as a reference point (0 mm), as the distance between the horizontal projection of the upper eyelid margin and palpebral rim while the eye is held manually from the eyelashes and pulled forward horizontally when the eye is in primary gaze. The clinical presence of eyelash ptosis was graded between 0 and 3.^21^ Dermatochalasis was defined as the presence of an excessive skin fold over the upper eyelid that may be accompanied by periorbital fat prolapse.

### Statistical Analysis

Statistical analysis was performed using SPSS version 18.0 (SPSS Inc., Chicago, IL) software pack. Comparisons between groups were analyzed using Student’s t-test for data with normal distribution and Mann-Whitney U test for data with non-normal distribution. Categorical variables were compared using chi-square test and Fisher’s exact test. A p value <0.05 was considered statistically significant.

## Results

The mean age of the patients was 49.9±11.4 (26-67) years in Group 1 and 50.6±8.9 (27-69) years in Group 2 (p=0.557). Group 1 showed statistically significant male predominance and significantly higher body mass index (BMI) when compared with Group 2 (Table 1).

The upper lid vertical and anterior distraction distances were 13.3±4.1 (6-27) mm and 7.4±2.1 (3-13.5) mm in Group 1, respectively, which were significantly greater than the distances in Group 2 (p<0.05). There were no significant differences in interpalpebral distance and levator function between the groups (p>0.05) (Table 2).

When the patients were compared in terms of periorbital alterations, dermatochalasis and eyelash ptosis were significantly more common in Group 1 (52.1% and 81.3% in Group 1, 27.3% and 22.7% in Group 2, respectively; p<0.05). It was observed that the degree of eyelash ptosis was also higher in patients with OSAS (p<0.05) (Table 3).

The prevalence of FES was calculated as 16.6% among all OSAS patients, and all patients with FES (n=8) were diagnosed as having severe OSAS. The comparison of demographic and clinical characteristics between patients with and without FES is shown in Table 4.

## Discussion

OSAS is a sleep disorder characterized by recurring upper respiratory tract obstructions and reduced oxygen saturation.^1^ The prevalence of moderate and severe OSAS is 2-6%, while this rate is about 14% for mild OSAS. The prevalence among patients referred to sleep clinics reaches 21-90%.^4^ In addition to associated symptoms such as loud snoring, episodes of apnea, and excessive daytime sleepiness, OSAS is also clinically important because it increases the severity and risk of life-threatening diseases (such as coronary artery disease, cerebrovascular events, atrial fibrillation, hypertension, neurocognitive disorders, and endocrine and metabolic diseases) that cause serious morbidity and mortality.^9^

FES is the most widely known ocular comorbidity of OSAS, and OSAS is also reported as the most common systemic disease associated with FES.^16^ The association between FES and OSAS was first identified by Woog^11^ FES is characterized by lax, easily foldable eyelids and papillary conjunctivitis.^12^ In previous studies, the prevalence of FES has varied between 2% and 32%.^22,26^ This may be due to the lack of standardized diagnostic criteria for FES and the use of subjective diagnostic methods.^22,23,24,25,26^ In studies reporting high FES rates, generally only upper eyelid laxity was considered, without evaluation of the presence of other findings associated with a diagnosis of FES.^19,23^ On the other hand, as both FES and OSAS are independently associated with obesity and male sex, it is difficult to say that OSAS is directly associated with FES.^27^ Beis et al.^28^ demonstrated that FES was associated with OSAS but not with obesity. In a large cross-sectional study by Ezra et al.^29^, FES was shown to be strongly correlated with OSAS and keratoconus. Wang et al.^30^ proposed in their meta-analysis that the prevalence of FES was higher in OSAS and its incidence increased with OSAS severity. In our study, the prevalence of FES among all OSAS patients was 16.6%. The FES prevalence determined in the present study is in concordance with the literature and all patients with FES (n=8) had severe OSAS.

Various qualitative and quantitative methods for the evaluation of eyelid laxity have been described in the literature. Liu et al.^31^ classified FES severity as grade 0 (no FES, no tarsal conjunctiva visible), grade 1 (less than one-third of the upper tarsal conjunctiva visible), grade 2 (one third to half of the upper tarsal conjunctiva visible), and grade 3 (more than half of the upper tarsal conjunctiva visible). McNab^22^ measured the vertical manual displacement of the lax upper eyelid in patients with FES and termed this the “vertical eyelid pull.” Robert et al.^19^ measured the maximum distance between the palpebral rim and pupil after vertical manual lid traction and termed this “vertical hyperlaxity.” Karger et al.^24^ calculated the force required for vertical displacement of the upper eyelid with a strain gauge device they developed. Mojon et al.^23^ evaluated eyelid displacement based on the lower lid laxity assessment described by Liu and Staisor in OSAS patients. Iyengar and Khan^20^ measured the anterior displacement of both upper lids in patients with symptomatic FES in one eye and asymptomatic FES in the other eye who were followed up for more than 5 years. They obtained measurements by manually holding the eyelashes and pulling forward horizontally and measuring the distance between the distracted eyelid and the corneal apex. The mean anterior distraction distance was 17.09 (14-20) mm in the symptomatic lids and 11.72 (10-15) mm in the asymptomatic lids. The mean difference between the two lids was statistically significant at 5.6 mm (p<0.02, t test). Considering the possibility that globe size could lead to inaccuracies in the measurement technique used by Iyengar and Khan^20^, in our study we calculated anterior distraction distance using the palpebral rim as a reference point (0 mm) and subtracting the distance between this point and the horizontal projection of the lid margin with the eye open in primary gaze from the distance measured when the eyelid was held manually from the eyelashes and pulled forward. Vertical distraction distance was determined based on the method described by Robert et al.^19^ According to these measurements, both anterior and vertical lid distraction distances in patients with OSAS were significantly higher than in patients with simple snoring. When only OSAS patients were evaluated, those with FES had higher distraction distances than those without FES. While this supports the presence of a certain amount of lid laxity in OSAS, it also suggests that it is more severe in the presence of FES. Moreover, the lax eyelid was associated with a lower amount of elastin in the tarsal tissue, while pathologic examination of uvula tissue from OSAS patients who underwent uvulopharyngoplasty also revealed loss of elastic fibers and elastin disorganization.^32,33^ Our findings are in parallel with this elastic tissue theory explaining the association between FES and OSAS. On the other hand, Fox et al.^34^ quantitatively evaluated eyelid laxity in bedside ophthalmological examinations of patients evaluated with PSG and reported that these markers (upper lid vertical traction, horizontal eyelid distraction, eyelash ptosis) were not associated with the presence and severity of OSAS. However, statistical analyses in the study were performed based on mean values obtained after grading the data between 0 and 4, and although not significant, the laxity measurements tended to increase as OSAS severity increased.

Langford and Linberg^35^ argued that eyelash ptosis and loss of eyelash alignment may be new signs of FES. Eyelash ptosis has been attributed to loss of tissue elasticity around the eyelashes. In our study, eyelash ptosis was more common among OSAS patients than patients with simple snoring. The mean eyelash ptosis grade was also significantly higher in the patients with OSAS. In addition, eyelash ptosis prevalence and severity were higher in OSAS patients with FES than in those without FES. Moreover, all FES patients showed substantial loss of eyelash alignment in addition to eyelash ptosis, whereas this was not seen in patients without FES. This can be interpreted as evidence that in addition to the presence of eyelash ptosis, the grade of eyelash ptosis and accompanying loss of eyelash alignment may be more specific for the diagnosis of FES. On the other hand, Malik et al.^21^ reported that eyelash ptosis accompanied a considerable proportion of cases of congenital and acquired blepharoptosis. They reported that grade 2 eyelash ptosis was detected in 28.9% of patients with acquired blepharoptosis and 6.7% of the control group, whereas eyelash ptosis of grade 1 or higher was found in 83.5% of patients with acquired blepharoptosis and 33.3% of the control group. In the present study, eyelash ptosis of grade 2 or higher was observed in 29.2% of patients with OSAS but was not detected in any patient with simple snoring. Among the OSAS group, this rate was 62.5% among those with FES and 12.5% among those without FES. Among the OSAS patients without FES, grade 1 eyelash ptosis was detected in 39.3% in addition to the presence of dermatochalasis. Therefore, it can be thought that the low-grade eyelash ptosis observed in OSAS patients without FES is caused by connective tissue laxity/increased elasticity in periorbital tissues, which is also associated with the accompanying dermatochalasis.

### Study Limitations

The strengths of the present study included that the patients were diagnosed with OSAS or simple snoring according to the results of PSG, the gold standard method, lid laxity was determined by quantitative measurements, and eyelash ptosis was objectively graded. The study limitations were that it was cross-sectional and included a small number of patients. For the moderate/severe OSAS group, the exclusion of patients with systemic diseases such as diabetes mellitus prevented pathological processes that may be associated with other systemic diseases from affecting the results. However, because the OSAS patients included in the study were in the early stages in terms of their systemic status and may not yet have developed systemic effects, their findings may have been less severe than expected. In addition, the male dominance and significantly higher BMI values in the OSAS group may also have been a source of bias in the results. More accurate data may be obtained from future studies that use the same objective evaluations in larger patient series and use regression analysis to rule out factors that may have a role.

## Conclusion

In conclusion, the present study determined that OSAS patients had greater eyelid laxity and significantly more frequent and severe eyelash ptosis. In ophthalmology practice, questioning patients with lax eyelids and especially eyelash ptosis about the typical symptoms for OSAS diagnosis and referring patients with those symptoms to sleep clinics seems potentially beneficial in terms of limiting the morbidity and mortality of the disease.

## Figures and Tables

**Table 1:**
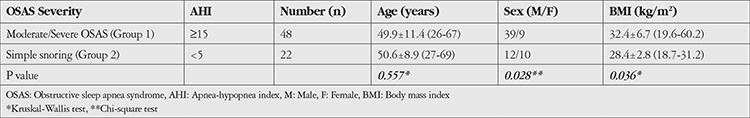
Demographic characteristics of the patients

**Table 2:**
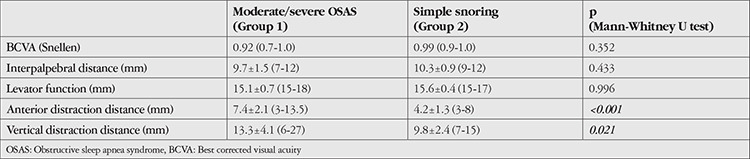
Comparison of best corrected visual acuity, lid function, and lid laxity of the patients

**Table 3:**
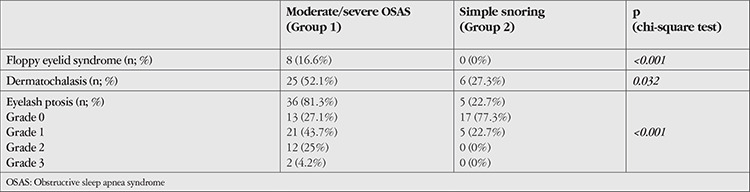
Comparison of periorbital tissue changes observed in the patient groups

**Table 4:**
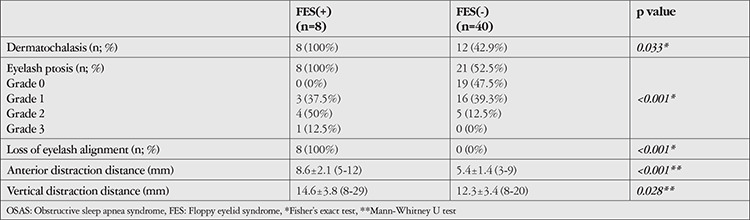
Comparison of periorbital tissue changes in OSAS patients with and without floppy eyelid syndrome (FES)
